# A Look into the Melting Pot: The *mec*C-Harboring Region Is a Recombination Hot Spot in *Staphylococcus stepanovicii*

**DOI:** 10.1371/journal.pone.0147150

**Published:** 2016-01-22

**Authors:** Torsten Semmler, Ewan M. Harrison, Antina Lübke-Becker, Rainer G. Ulrich, Lothar H. Wieler, Sebastian Guenther, Ivonne Stamm, Anne-Merethe Hanssen, Mark A. Holmes, Szilvia Vincze, Birgit Walther

**Affiliations:** 1 Robert Koch Institute, Berlin, Germany; 2 Department of Veterinary Medicine, University of Cambridge, Cambridge, United Kingdom; 3 Institute of Microbiology and Epizootics, Veterinary Faculty, Freie Universität Berlin, Berlin, Germany; 4 Institute for Novel and Emerging Infectious Diseases, Friedrich-Loeffler-Institut, Federal Research Institute for Animal Health, Greifswald-Insel Riems, Germany; 5 Vet Med Labor GmbH, Division of IDEXX Laboratories, Ludwigsburg, Germany; 6 Department of Medical Biology, Faculty of Health Sciences, UiT – The Arctic University of Norway, Tromsø, Norway; University of Mississippi Medical Center, UNITED STATES

## Abstract

**Introduction:**

Horizontal gene transfer (HGT) is an important driver for resistance- and virulence factor accumulation in pathogenic bacteria such as *Staphylococcus aureus*.

**Methods:**

Here, we have investigated the downstream region of the bacterial chromosomal attachment site (*attB*) for the staphylococcal cassette chromosome *mec* (SCC*mec*) element of a commensal *mecC*-positive *Staphylococcus stepanovicii* strain (IMT28705; ODD4) with respect to genetic composition and indications of HGT. *S*. *stepanovicii* IMT28705 was isolated from a fecal sample of a trapped wild bank vole (*Myodes glareolus*) during a screening study (National Network on “Rodent-Borne Pathogens”) in Germany. Whole genome sequencing (WGS) of IMT28705 together with the *mecC*-negative type strain CM7717 was conducted in order to comparatively investigate the genomic region downstream of *attB* (GenBank accession no. KR732654 and KR732653).

**Results:**

The bank vole isolate (IMT28705) harbors a *mecC* gene which shares 99.2% nucleotide (and 98.5% amino acid) sequence identity with *mecC* of MRSA_LGA251. In addition, the *mecC*-encoding region harbors the typical *blaZ-mecC-mecR1-mecI* structure, corresponding with the class E *mec* complex. While the sequences downstream of *attB* in both *S*. *stepanovicii* isolates (IMT28705 and CM7717) are partitioned by 15 bp direct repeats, further comparison revealed a remarkable low concordance of gene content, indicating a chromosomal “hot spot” for foreign DNA integration and exchange.

**Conclusion:**

Our data highlight the necessity for further research on transmission routes of resistance encoding factors from the environmental and wildlife resistome.

## Introduction

Since the late 1970’s, methicillin resistance in coagulase-positive staphylococci (CPS) emerged as a major threat to both human and veterinary medicine [1]. Methicillin resistance is conferred by an additional penicillin binding protein (PBP2a), which substitutes the transpeptidase function of the native PBP2 during the crucial process of bacterial cell wall building in the presence of beta-lactam antibiotics [2]. The gene encoding PBP2a in staphylococci is part of a *mec* complex, consisting of a methicillin resistance encoding *mec* homologue (*mec*A or *mec*C), which is usually accompanied by intact or truncated versions of the regulatory genes *mec*I (repressor) and *mec*R1 (sensor inducer) [3,4]. A further regulatory component, *mec*R2 (antirepressor) influencing methicillin resistance levels, was recently described [5]. So far, three different allotypes were described for *mec*A and *mec*C, respectively [6].

The *mec* complex can be part of a larger, potential mobile element called staphylococcal cassette chromosome *mec* (SCC*mec*). These SCC*mec* elements also contain site-specific recombinase genes (*ccr*AB or *ccr*C) and flanking “junkyard” or “joining” (“J”) regions (J1–J3) [7,8]. In *Staphylococcus aureus*, the SCC*mec* insertion occurs at the bacterial chromosomal attachment site (*att*BSCC), represented by the terminal nucleotides of the 3’ end of an rRNA methyltransferase (formerly: *orf*X), leaving the gene functionally intact [9]. Usually, SCC*mec* elements are flanked by characteristic sequences of 15 bp (“direct repeats”; DR) that are recognized by the recombinases catalyzing the processes of chromosomal excision and integration [10,11]. Spontaneous excision of SCC*mec* leaves only one DR in the chromosome [12,13]. A review by the International Working Group on the Staphylococcal Cassette Chromosome elements (IWG-SCC) published in 2009 provides an overview on the eleven distinct SCC*mec* elements, several other SCC’s as well as atypical SCC*mec* elements which have been described so far [14]. In addition, the IWG-SCC hosts a data base on current SCC*mec* elements (http://www.sccmec.org/).

In 2011, a *mecA* homologue denominated as *mecC* located on an SCC*mec* type XI element was described in methicillin resistant *Staphylococcus aureus* (MRSA) isolates from both human and bovine origin, and later also from a range of other animal species, including companion animals and wild small mammals [15–19]. This *mec*C gene shows 69% nucleotide- and 63% amino acid sequence identity to *mec*A resp. PBP2a of *S*. *aureus* N315. Similar to *mec*A, it was the suspect that *mecC* also originated from coagulase-negative staphylococci (CNS). The recent findings of two further *mec*C allotypes in CNS denominated as *mec*C1 (*S*. *xylosus*, cattle) and *mec*C2 (*S*. *saprophyticus*, common shrew) may support this hypothesis [20,21]. In addition, a further study identified the *mec*C gene in a CNS isolate from an Eurasian lynx which is most probably *Staphylococcus stepanovicii* [22], a species that is considered as a persistent member of physiological microflora of the skin of wild small mammals [23]. Here we report the characterization of the methicillin resistance encoding region in a *S*. *stepanovicii* isolate (IMT28705; ODD4) harboring a *mec*C gene. According to the definition of the IWG-SCC [14], the region was denominated as ψSCC*mec*IMT28705. The *mec*C-negative *S*. *stepanovicii* type strain CCM7717[24] was included to compare the genomic arrangement of the two regions downstream of *att*BSCC.

## Material and Methods

The *Staphylococcus stepanovicii* strain IMT28705 (ODD4) was isolated in August 2011 from a fecal sample of a live-trapped male bank vole (*Myodes glareolus*) of 26 g weight, collected during October 2011 at forest monitoring site #1 in Jeeser, Mecklenburg-Western Pomerania, North-East Germany [25], as part of a screening study focusing on pathogens from wild rodents in Germany (Network “Rodent-Borne Pathogens” [26]. The rodent trapping was approved by the competent authority of the Federal State of Mecklenburg-Western Pomerania, Germany, on the basis of national and European legislation (LALLF-M-V/TSD/7221.3-030/09). Rectal swabs were enriched by use of an enrichment broth to enhance staphylococcal growth and to prevent Gram-negative overgrowth [27]. A positive PCR-result for the *mec*C gene using the primers published by Cuny et al. [15] was the initial reason for sequencing the whole genome of the *mec*C-positive strain (IMT28705) on a HiSeq (Illumina, USA). To gain deeper insights into the genomic region downstream of *att*BSCC within this staphylococcal species, we included the *mec*C-negative *S*. *stepanovicii* type strain (CCM7717) [24]. The reads were assembled using CLC Genomics Workbench 7.5 (CLC bio, Denmark) and open reading frames (ORFs) were predicted using Prodigal: Prokaryotic Dynamic Programming Genefinding Algorithm [28]. Annotation of ORFs and prediction of (protein) coding sequences (CDS) was performed by The RAST Server: rapid annotations using subsystems technology [29]. Putative CDS function and conserved domains were predicted with blastn and blastx using the NCBI database (http://blast.ncbi.nlm.nih.gov/Blast.cgi). The presence of sequence homology to proteins encoded by diverse families of transposable elements in the genomes was performed by PSI-Blast within the TransposonPSI tool (http://transposonpsi.sourceforge.net/). For genomic comparative analyses Geneious 7.1.5 was employed. Putative integration site sequences were identified as DR using the following screening sequence: GAA[AG][CG][TA]TATCATAA[GA].

Both *S*. *stepanovicii* isolates were subjected to disc diffusion test using cefoxitin (30 μg) according to CLSI standards [30,31]. Automated determination of minimum inhibitory concentrations (MIC) for both *S*. *stepanovicii* isolates was performed using the bioMerieux VITEK^®^2 system according to the manufacturer’s instructions, including benzyl-penicillin, oxacillin, gentamicin, enrofloxacin, marbofloxacin, erythromycin, clindamycin, tetracycline, chloramphenicol and trimethoprim-sulfamethoxazole according to the Clinical and Laboratory Standards Institute.

## Results and Discussion

**Fig 1:**
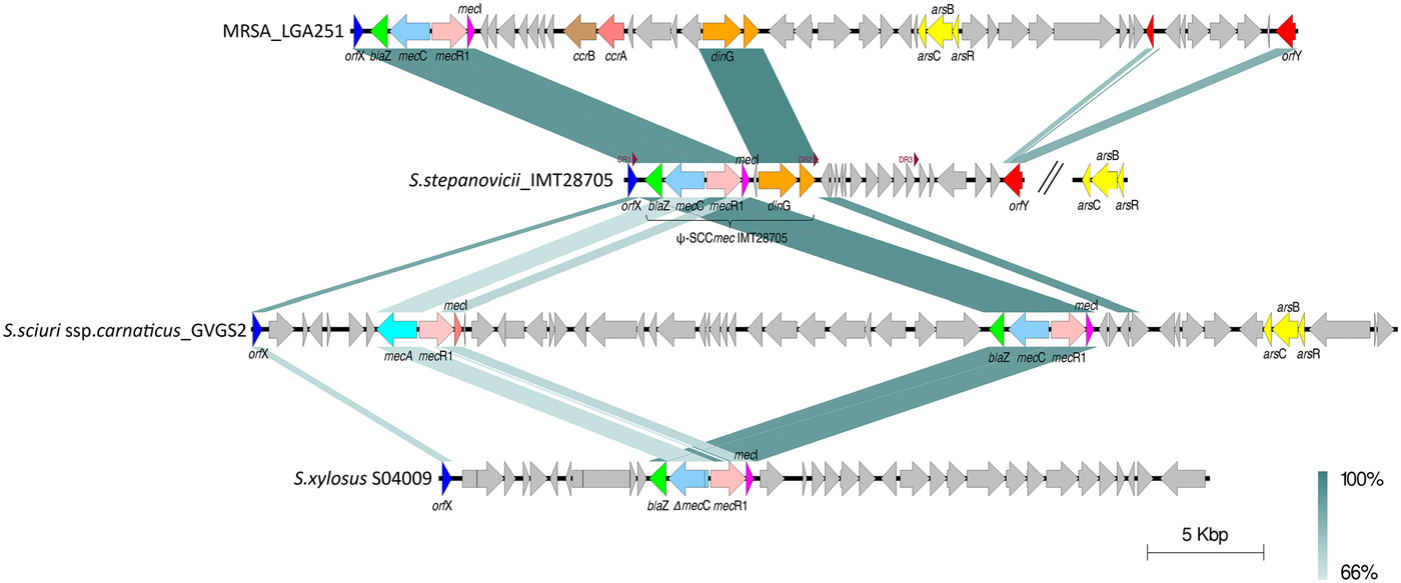
Comparison of the genetic structure of the region downstream of *orf*X-like gene (rRNA-methyltransferase) for *S*. *stepanovicii* strain IMT28705 with MRSA_LGA251, *S*. *sciuri* ssp. *carnaticus*_GVGS2, and *S*. *xylosus*_SO4009. Direct repeats (DR) of IMT28705 are indicated by red arrowheads. Conserved DNA regions are shown by dark green color; more dissimilar sequences are indicated with light green. Arrows indicate ORFs and their orientation on the genome. Selected ORFs were colored to illustrate orthologs between the genomes. Nucleotide sequence similarities for IMT28705 are given in Table 1.

**Fig 2:**
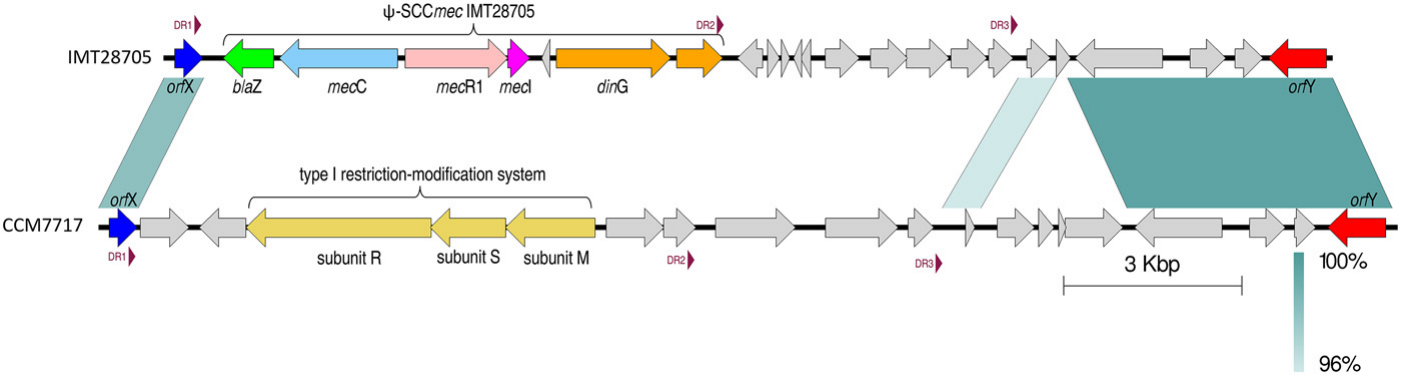
Genomic comparison of the DNA sequences between *orf*X (rRNA-methyltransferase-like gene) and the *orf*Y-like gene of the *S*. *stepanovicii* strains IMT28705 (*mec*C-positive) with CCM7717 (*mec*C-negative). Direct repeats (DR) are indicated by red arrowheads, conserved DNA regions are shown by dark green color; more dissimilar sequences are indicated with light green. A remarkable divergence in the genomic structure downstream of the bacterial chromosomal attachment site (*att*B) is obvious, indicating a genomic “hot spot” for integration and excision of foreign DNA in *S*. *stepanovicii*. In addition, a blastn search (12/2014) revealed an absolutely low degree of nucleotide sequence similarities for this region of CCM7717 (S1 Table).

Species identity of IMT28705 was determined by 16S rDNA sequence analysis (GenBank accession no. KR732655), revealing a homology of 99.9% with the type strain CCM7717. Here we report on the entire nucleotide sequence region between the rRNA-methyltransferase (*orf*X)-like gene and the tRNA dihydrouridine synthase B (*orf*Y)-like gene in a *mec*C-positive strain (IMT28705, GenBank accession no. KR732654) and the *mec*C-negative reference strain (CCM7717, GenBank accession no. KR732653). Genome sequencing revealed that strain IMT28705 harbors a *mec*C gene which shares 99.2% nucleotide (and 98.5% amino acid) sequence identity with *mec*C of MRSA_LGA251. At the chromosomal insertion site of SCC*mec*, five terminal amino acids were encoded by 15 bp, followed by a stop codon sequence (TGA). Insertion of SCC*mec* alters this site to *att*R1 or DR1. In IMT28705, the *att*R1 integration site at the end of the rRNA-methyltransferase (*orf*X)-like gene is indicated by GAAAGTTATCATAAA**TGA** (DR1) encoding the terminal amino acids ESYHK. Corresponding DRs are located 8,884 bp (DR2: GAAGCATATCATAAA**TGA**, encoding EAYHK) as well as 13,757 bp (DR3: GAAAGTTATCATAAG**TGA**, encoding again ESYHK) downstream of DR1 (Fig 1). These DRs are analogues sequences to those reported for other SCC*mec* elements, including those reported for MRSA [11,32], indicating a broad and general exchangeability of genomic regions flanked by these universal distributed DR sequences downstream of *att*BSCC, at least among staphylococci. Furthermore, these DR sequences were also detected as partitioning sequences in prototype strain CCM7717, starting with the terminal EAYHK-encoded motif within the rRNA-methyltransferase (*orf*X)-like gene (Fig 2).

In IMT28705, the *mec*C-encoding region follows directly the *att*R1 (DR1) integration site with a very short (25 bp) J-region. Immediately downstream follows the typical *bla*Z-*mec*C-*mec*R1-*mec*I structure of 5,163 bp corresponding to the class E *mec* complex described for MRSA_LG251, *S*. *xylosus* (S04009) *and S*. *sciuri* ssp. *carnaticus* [16,20,33] (Fig 1). A similar structure (including *mec*B instead of different *mec*C allotypes) was reported for *Macrococcus caseolyticus* (a Gram-positive species that was formerly classified as *Staphylococcus caseolyticus*), either as part of a transposon located on plasmids or within an SCC*mec* element [34]. A comparative analysis of these *mec*-encoding regions including *M*. *caseolyticus* is provided in Table 1.

**Table 1:** Comparison of IMT28705 class E *mec* complex with other *mec* E complexes.

	Nucleotide sequence similarity		MRSA_LGA251		*S*. *stepanovicii*_3orsfiwi		*S*. *xylosus*_S04009		*S*. *sciruri carnaticus*_GVGS2		*M*. *caseolyticus*_JCSC7096	
	ORF	Description / presumptive function	C	NI	C	NI	C	NI	C	NI	C	NI
	*bla*Z	Beta-lactamase	100%	97%	87%	99%	98%	91%	100%	98%	98%	61%
	*mec*C	Penicillin-binding protein PBP2a, transpeptidase	100%	99%	88%	100%	99%*	93%	100%	96%	100%	63%
**ψSCC*mec***_**IMT28705**_	*mec*R	Methicillin resistance regulatory sensor-transducer MecR1	100%	89%	n. a.	n. a.	100%	97%	100%	90%	99%	58%
	*mec*I	Methicillin resistance repressor MecI	95%	91%	n. a.	n. a.	100%	99%	98%	91%	100%	64%
	CDS_1	hypothetical protein	69%	91%	n. a.	n. a.	33%	94%	n. a.	n. a.	n. a.	n. a.
	*din*G	DinG family (ATP-dependent helicase YoaA)	100%	96%	n. a.	n. a.	n. a.	n. a.	n. a.	n. a.	n. a.	n. a.
	CDS_2	hypothetical protein	100%	99%	n. a.	n. a.	n. a.	n. a.	n. a.	n. a.	n. a.	n. a.

Abbreviations: ORF: open reading frame; C: coverage; N: nucleotide identity, *pseudogene, CDS: coding sequence; Nucleotide sequence identities (%) of homologues genes of different *mec* E complexes (GenBank entries) in comparison with ψSCC*mec*_IMT28705_. Notably, nucleotide sequence identities for the regulatory genes *mec*R1 and *mec*I are 97% and 99% for *S*. *xylosus*_S04009 and thus higher than those for MRSA_LGA2951 (89% / 91%) and *S*. *sciruri carnaticus*_GVGS2 (90%/91%).

It has been assumed that the initial formation of the *mec* complex is a result of the integration of a (putative chromosomal) *mec* allotype in an intact beta-lactamase operon *bla*Z-(*mec*A)-*bla*R1-*bla*I followed by the loss of the native beta-lactamase-encoding *bla*Z over time [34,35]. Furthermore, the *bla*Z regulator genes *bla*R1 and *bla*I influence the expression levels regulated by *mec*R1 and *mec*I (“cross-talk”) of *mec*A in methicillin resistant staphylococci, too [5]. From an evolutionary perspective, the *mec* E with its “ancestral” *bla*Z is an interesting phenomenon: This structure is either more conserved than comparable progenitor forms of other *mec* complexes harboring *mec*A allotypes and/or benefits from the potential antimicrobial activity of *bla*Z. The penicillin resistance noticed for an oxacillin susceptible *S*. *xylosus* strain harboring a truncated *mec*C1 within its *mec* E complex might be an example for the latter case [20]. Contrariwise, *mec*C MRSA strains did not express elevated beta-lactamase activity levels so far [6].

For the *S*. *stepanovicii* strains reported here, the disc diffusion test showed an inhibition zone diameter of 22 mm for cefoxitin for IMT28705 (= methicillin resistant phenotype), the *mec*C-negative strain (CCM7717) exhibited 29 mm (= susceptible phenotype) according to the utilized interpretation criteria [30]. As presented in Table 2, the oxacillin MICs for both the *mec*C-negative *S*. *stepanovicii* strain and the *mec*C-positive strain IMT28705 were ≥4 mg/L. However, increased oxacillin MICs for CNS lacking *mec*A or *mec*C as well as unusual beta-lactamase (hyper-) production were reported before, especially for isolates from bovine milk samples [36,37]. Moreover, Skov et al. reported in 2014 that cefoxitin is more reliable than oxacillin for *mec*C-associated methicillin resistance in *S*. *aureus* [38].

**Table 2:** Results of minimum inhibitory concentration (MIC) testing of *S*. *stepanovicii* isolates using VITEK^®^2.

Antimicrobial substance	IMT28705	CM7717
benzyl-penicillin	**≥ 0.5**	0.12
oxacillin	**≥ 4**	**≥ 4**
gentamicin	≤ 0.5	≤ 0.5
enrofloxacin	≤ 0.5	≤ 0.5
marbofloxacin	1	≤ 0,5
erythromycin	≤ 0.25	≤ 0.25
clindamycin	≤ 0.25	≤ 0.25
tetracycline	≤ 1	≤ 1
chloramphenicol	8	8
trimethoprim-sulfamethoxazole	≤ 10	≤ 10
Cefoxitin-Screen	+	-
Inducible clindamycin resistance	-	-

Bold = resistant according to Vet01-S2 [30].

No major differences were seen in MICs for the other antibiotics tested (Table 2).

In IMT28705, the region between the *orf*X and *orf*Y-like genes does not harbor a known transposable element, neither as a full ORF nor as a truncated remnant (Table 3 and Fig 1). In addition, no *ccr* homologues were identified. Thus, the *mec*C region in IMT28705 seems to lack factors associated with potential mobility of an SCC*mec* element. We therefore propose to denominate the region between DR1 and DR2 (8,884 bp) lacking *ccr* genes as ψSCC*mec*_IMT28705_ (Fig 1), according to the definition provided by the IWG-SCC [14].

**Table 3:** Nucleotide sequence similarities downstream of *rlm*H (formerly *orf*X) to *orf*Y-like genes (gb KR732654) of the bank vole-derived *mec*C-positive *S*. *stepanovicii* strain IMT28705 and other bacterial strains taken from GenBank entries.

ORF	Description / presumptive function	from*	to*	bp	C	NI	Accession no.	Species (gene location), strain
1	LSU m3Psi1915 methyltransferase RlmH (*orf*X-like)	219	698	480	100%	87%	HG515014.1	*S*. *sciuri* ssp. *carnaticus*, GVGS2
	**ψSCC*mec***_**IMT28705**_	**1,039**	**9,453**	**8,884**				
2	Beta-lactamase (EC 3.5.2.6)	1,039	1,890	852	100%	98%	HG515014.1	*S*. *sciuri* ssp. *carnaticus*, GVGS2
3	Penicillin-binding protein PBP2a, transpeptidase	1,984	3,978	1,995	100%	99%	FR821779.1	*S*. *aureus* (*SCCmec*), MRSA_LGA251
4	Methicillin resistance regulatory sensor-transducer MecR1	4,100	5,833	1,734	100%	97%	HE993884.1	*S*. *xylosus* (SCCmec), HE993884
5	Methicillin resistance repressor MecI	5,830	6,207	378	100%	99%	HE993884.1	S. xylosus (SCC*mec*), HE993884
6	hypothetical protein 1	6,403	6,543	141	69%	91%	FR821779.1	*S*. *aureus* (SCC*mec*), MRSA_LGA251
7	DinG family ATP-dependent helicase YoaA	6,649	8,580	1,932	100%	96%	FR821779.1	*S*. *aureus* (SCC*mec*), MRSA_LGA251
8	putative membrane protein	8,674	9,453	780	100%	99%	FR821779.1	*S*. *aureus* (SCC*mec*),MRSA_ LGA251
	**Region between DR2 and DR3**	**9,580**	**14,455**	**4,875**				
9	PhnB, putative ribosomal methyltransferase	9,701	10,129	429	100%	93%	KF527883.1	*S*. *aureus*, (*SCCmec*), NTUH-4729
10	hypothetical protein (transcriptional regulator DeoR family)	10,208	10,411	204	98%	93%	HG515014.1	*S*. *sciuri* ssp. *carnaticus*, (SCC*mec*), GVGS2
11	hypothetical protein (transcriptional regulator DeoR family)	10,439	10,582	144	97%	100%	|HG515014.1	*S*. *sciuri* ssp. *carnaticus*, (SCC*mec*), GVGS3
12	hypothetical protein	10,643	10,783	141				no similarities
13	hypothetical protein	10,764	10,940	177				no similarities
14	hypothetical protein	11,181	11,756	576	88%	72%	CP002439.1	*S*. *pseudintermedius*, (SCC*mec*), HKU10-03
15	conserved hypothetical protein	11,939	12,565	627	100%	89%	AB498756.1	*M*. *caseolyticus*, (*mec*B region), JCSC7096
16	putative glucose-1-phosphate adenylyltransferase GlgD	12,543	13,265	723	100%	96%	AB498757.1	*M*. *caseolyticus*, *(mec*B region), JCSC7528
17	putative serine/threonine-specific protein phosphatase 2^1^	13,296	13,904	609				no similarities
18	hypothetical protein	13,930	14,343	414	100%	87%	AB261975.1	*S*. *aureus* (J1 region, SCC*mec*), RN7170
	**Region between DR3 and *orf*Y-like gene**	**14,456**	**18,645**	**4,189**				
19	hypothetical protein	14,577	14,975	399				no similarities
20	hypothetical protein	15,071	15,316	246				no similarities
21	hypothetical protein	15,391	16,866	1,476	46%	66%	HE980450.1	*S*. *aureus*,(SCC*mec*), M06/0171
22	hypothetical protein	17,323	17,928	606	92%	69%	CP006044.1	*S*. *aureus* (*SCCmec*), CA-347
23	putative acetyltransferase (GNAT) family protein	18,083	18,556	474	85%	76%	CP007447.1	*S*. *aureus* (SCC*mec*), XN108
24	probable tRNA-dihydrouridine synthase (*orf*Y-like)	18,645	19,625	981	98%	85%	FR821777.2	*S*. *aureus* (SCC*mec*), MSHR1132

Abbreviations: ORF: open reading frame, bp: base pair, C: Coverage; NI: nucleotide similarity; DR: direct repeat of 15 bp

^1^ predicted by use of blastx;

* nucleotide position in **ψSCC*mec***_**IMT28705**_ (GenBank accession no. KR732654)

Downstream of the class E *mec* complex, the *damage inducible gene* G (*din*G) of the *S*. *stepanovicii* strain IMT28705 shares 96% nucleotide sequence identity with the corresponding homologue of MRSA_LGA251 (Fig 1, Table 3). The *din*G encoded protein represents a fusion between a helicase and a nuclease often working together in the processes involved in DNA repair and recombination [39], a function providing a potential benefit in regions associated with recombination events. The last ORF (780 bp) comprised by ψSCC*mec*_IMT28705_ shows 96% nucleotide sequence identity to a hypothetical membrane protein (SARLGA251_00430) of MRSA_LGA251, lacking putative conserved regions (Table 3). Insertion sequences (IS) and transposons (Tn), frequently associated with SCC*mec* elements such as IS431, IS1272 or Tn554 were not identified. A second region comprising 4,876 bp in IMT28705 is flanked by DR2 and DR3 and starts with an ORF (429 bp) encoding a putative PhnB-like protein, which was reported for SCC*mec*V elements of Indian origin only recently, including structural folds similar to bleomycin resistance protein [40]. Next, two putative transcriptional regulators (DeoR family) also present in *S*. *sciuri* ssp. *carnaticus*_GVGS2, (Table 3) as well as in many other staphylococci, are part of the region downstream of *orf*X (data not shown). Two further ORFs providing no significant similarities in the NCBI nucleotide / protein databases were followed by a coding region for a hypothetical protein also present in the SCC*mec* element of *S*. *pseudintermedius* strain HKU-1003. These are adjacent to sequences encoding a conserved hypothetical protein and a putative glucose-1-phosphate adenylyltransferase, both reported for the *mec*B ecoding region in *M*. *caseolyticus*. The next ORFs encode a putative serine/threonine-specific protein phosphatase (predicted by use of blastx) and a further hypothetical protein also described for the J1 region in MRSA strain RN71170 (Table 3).

The third region flanked by DR3 and the *orf*Y-like gene comprises 4,171 bp and at least six distinct ORFs. Notably, a search on the EMBL database for sequences of this region (between bp positions 14,577 and 16,866) showed only a few hits and a very low nucleotide sequence similarity (date: 12/2014) by use of blastn (Table 3). An arsenic resistance operon described for the SCC*mec* region of MRSA_LGA251 is located 35 kb downstream of the *orf*Y-like gene in IMT28705. A similar operon is reported for *S*. *xylosus*_S04009 (Fig 1).

The *mec*C-negative reference strain (CCM7717) showed DR1 sequence GAAGCATATCATAAA**TAA** at the 3’ end of the rRNA-methyltransferase (*orf*X-like) gene, followed by a region of 9,527 bp ending with DR2 (GAAAGTTATCATAAG**TAA**) and a further part consisting of 4,057 bp ending with DR3 (GAAAGTTATCATAAG**TGA**). As displayed in Fig 2, the gene content of this region differs remarkably from that of IMT28705. However, a type I restriction modification system (5,885 bp) consisting of *hsd*R, *hsd*S and *hsd*M is present downstream of *orf*X in CCM7717, showing significant nucleotide sequence similarities (coverage: 82%; identity 89%) with the homologous region in MRSA_LGA251 (S1 Table). This particular restriction modification system was described for SCC*mec*V and has been discussed as a stabilizing factor for these elements [10,32]. A further blastn search of the entire region downstream of DR1 in CCM7717 revealed some sequence similarities to ORFs with predominantly unknown functions within other staphylococci (especially in different SCC*mec* elements), but for seven ORFs the entered sequence data provided no significant hits or similarities so far (01/2015, S1 Table).

In recent years, it has been assumed that the methicillin resistance conferring PBP2a encoded by allotypes of *mec*A and *mec*C originates from CNS including the *S*. *sciuri* group, *Staphylococcus fleurettii* and other CNS [13,41,42]. The identification of the origin of genes encoding methicillin resistance among CNS is important for understanding the evolution of pathogenic methicillin resistant CPS and may contribute to the development of more effective control measures [13].

Here we report about a class E *mec* complex and some associated ORFs with strong similarities to the strains MRSA_LGA251 and *S*. *xylosus* S04009. Furthermore, the mosaic structure of the region downstream of *orf*X in both *S*. *stepanovicii* strains (IMT28705 and CCM7717) is not associated with the presence/or absence of *mec*C and/or an SCC*mec* element. Taken together, the *orf*X-like region seems to be a putative integration site of foreign DNA in *S*. *stepanovicii*, representing a “melting pot”, like it has been described for *S*. *aureus* and other members of the *Staphylococcus* genus previously [43,44]. Furthermore, a recent study revealed that the limited distance between the region downstream of *orf*X (recently renamed as *rlm*H) and the origin of replication (*ori*C) in *S*. *aureus* (38 kb in the genomes of IMT28705 and CCM7717) is a strong predictor for a recombination hot spot [45].

However, the broad distribution of certain genes within this particular chromosomal location in different staphylococcal species raises the question if other routes of horizontal gene transfer beside interaction with serine recombinase family genes (*ccr*) might exist. For instance, recent studies showed the general transmissibility of SCC*mec* elements or parts of them by different bacteriophages [46,47].

Whether ψSCC*mec*_IMT28705_ represents an evolutionary precursor of SCC*mec*XI or not is not clear yet, but is at least a possible option, which has been discussed for *mec*A-harboring CNS and MRSA before [34,35,44]. Further genomic analysis of CNS originating from wildlife (including small mammal rodents) is clearly needed to unravel the primordial origin of *mec*C (and other *mec* allotypes) and to answer the question where the *mec* complex gets its mobility from. In conclusion, our data highlight the role of the environmental and wildlife resistome as an important source of antibiotic resistance in opportunistic and zoonotic bacterial species such as *S*. *aureus* and the putative transferability of further factors and elements flanked by DR downstream of *att*B, an important “melting pot” for genetic rearrangements.

## Transparency Declarations

Dr. Stamm is an employee of IDEXX Vet Med Labor GmbH (Ludwigsburg). Whilst this author is a company employee, this does not alter the adherence to all the PLoS ONE policies on sharing data and materials for this manuscript. All other authors have declared that no competing interests exist.

## Supporting Information

S1 Table Nucleotide sequence similarities downstream of *orf*X (*rlm*H) to *orf*Y-like genes (gb KR732653) of the *mec*C-negative *S*. *stepanovicii* type strain (CCM7717) with other bacterial strains taken from GenBank entries. Abbreviations: ORF: open reading frame, bp: base pair, C: Coverage; NI: nucleotide sequence identity; DR: direct repeat of 15 bp. ^1^ predicted by use of blastx.
(DOCX)

## References

[1] Wieler LH, Ewers C, Guenther S, Walther B, Lübke-Becker A. Methicillin-resistant staphylococci (MRS) and extended-spectrum beta-lactamases (ESBL)-producing Enterobacteriaceae in companion animals: nosocomial infections as one reason for the rising prevalence of these potential zoonotic pathogens in clinical samples. Int J Med Microbiol. 2011;301(8):635–41. 10.1016/j.ijmm.2011.09.009 22000738

[2] Pinho MG, de Lencastre H, Tomasz A. An acquired and a native penicillin-binding protein cooperate in building the cell wall of drug-resistant staphylococci. Proc Natl Acad Sci USA 98. 2001;98(19):10886–91. 10.1073/pnas.191260798 PMC58569 11517340

[3] Ito T, Hiramatsu K, Tomasz A, de Lencastre H, Perreten V, Holden MT, et al Guidelines for reporting novel *mec*A gene homologues. Antimicrob Agents Chemother. 2012;56(10):4997–9. 10.1128/AAC.01199-12 22869575 PMC3457410

[4] Shore AC, Coleman DC. Staphylococcal cassette chromosome *mec*: recent advances and new insights. Int J Med Microbiol. 2013;303(6–7):350–9. 10.1016/j.ijmm.2013.02.002 23499303

[5] Arede P, Milheirico C, de Lencastre H, Oliveira DC. The anti-repressor MecR2 promotes the proteolysis of the mecA repressor and enables optimal expression of beta-lactam resistance in MRSA. PLoS pathogens. 2012;8(7):e1002816 10.1371/journal.ppat.1002816 22911052 PMC3406092

[6] Becker K, Ballhausen B, Köck R, Kriegeskorte A. Methicillin resistance in Staphylococcus isolates: the "mec alphabet" with specific consideration of mecC, a mec homolog associated with zoonotic S. aureus lineages. Int J Med Microbiol. 2014;304(7):794–804. 10.1016/j.ijmm.2014.06.007 25034857

[7] Deurenberg RH, Vink C, Oudhuis GJ, Mooij JE, Driessen C, Coppens G, et al Different clonal complexes of methicillin-resistant *Staphylococcus aureus* are disseminated in the Euregio Meuse-Rhine region. Antimicrob Agents Chemother. 2005;49(10):4263–71. 16189107 10.1128/AAC.49.10.4263-4271.2005 PMC1251497

[8] Ito T, Okuma K, Ma XX, Yuzawa H, Hiramatsu K. Insights on antibiotic resistance of *Staphylococcus aureus* from its whole genome: genomic island SCC. Drug Resist Updat. 2003;6(1):41–52. 12654286 10.1016/s1368-7646(03)00003-7

[9] Boundy S, Safo MK, Wang L, Musayev FN, O'Farrell HC, Rife JP, et al Characterization of the Staphylococcus aureus rRNA methyltransferase encoded by orfX, the gene containing the Staphylococcal Chromosome Cassette mec (SCCmec) insertion site. J Biol Chem. 2013;288(1):132–40. 10.1074/jbc.M112.385138 23150671 PMC3537007

[10] Hanssen AM, Ericson Sollid JU. SCC*mec* in staphylococci: genes on the move. FEMS Immunol Med Microbiol. 2006;46(1):8–20. 10.1111/j.1574-695X.2005.00009.x 16420592

[11] Wang L, Safo M, Archer GL. Characterization of DNA sequences required for the CcrAB-mediated integration of staphylococcal cassette chromosome *mec*, a *Staphylococcus aureus* genomic island. J Bacteriol. 2012;194(2):486–98. 10.1128/JB.05047-11 22056931 PMC3256654

[12] Katayama Y, Ito T, Hiramatsu K. Genetic organization of the chromosome region surrounding *mec*A in clinical staphylococcal strains: role of IS431-mediated *mec*I deletion in expression of resistance in *mec*A-carrying, low-level methicillin-resistant *Staphylococcus haemolyticus*. Antimicrob Agents Chemother. 2001;45(7):1955–63. 10.1128/AAC.45.7.1955-1963.2001 11408208 PMC90585

[13] Tsubakishita S, Kuwahara-Arai K, Sasaki T, Hiramatsu K. Origin and molecular evolution of the determinant of methicillin resistance in staphylococci. Antimicrob Agents Chemother. 2010;54(10):4352–9. 10.1128/AAC.00356-10 20679504 PMC2944575

[14] International Working Group on the Classification of Staphylococcal Cassette Chromosome (SCC) Elements” (IWG-SCC). Classification of staphylococcal cassette chromosome mec (SCCmec): guidelines for reporting novel SCCmec elements. Antimicrob Agents Chemother. 2009;53(12):4961–7. 10.1128/AAC.00579-09 19721075 PMC2786320

[15] Cuny C, Layer F, Strommenger B, Witte W. Rare occurrence of methicillin-resistant Staphylococcus aureus CC130 with a novel mecA homologue in humans in Germany. PloS one. 2011;6(9):e24360 10.1371/journal.pone.0024360 21931689 PMC3169590

[16] Garcia-Alvarez L, Holden MT, Lindsay H, Webb CR, Brown DF, Curran MD, et al Meticillin-resistant *Staphylococcus aureus* with a novel *mec*A homologue in human and bovine populations in the UK and Denmark: a descriptive study. Lancet Infect Dis. 2011;11(8):595–603. S1473-3099(11)70126-8 [pii] 10.1016/S1473-3099(11)70126-8 21641281 PMC3829197

[17] Gomez P, Gonzalez-Barrio D, Benito D, Garcia JT, Vinuela J, Zarazaga M, et al Detection of methicillin-resistant Staphylococcus aureus (MRSA) carrying the mecC gene in wild small mammals in Spain. J Antimicrob Chemother. 2014;69(8):2061–4. 10.1093/jac/dku100 24710026

[18] Paterson GK, Larsen AR, Robb A, Edwards GE, Pennycott TW, Foster G, et al The newly described *mec*A homologue, *mec*ALGA251, is present in methicillin-resistant *Staphylococcus aureus* isolates from a diverse range of host species. J Antimicrob Chemother. 2012;67(12):2809–13. 10.1093/jac/dks329 22941897 PMC3494845

[19] Walther B, Wieler LH, Vincze S, Antao EM, Brandenburg A, Stamm I, et al MRSA variant in companion animals. Emerg Infec Dis. 2012;18(12):2017–20. 10.3201/eid1812.120238 23171478 PMC3557870

[20] Harrison EM, Paterson GK, Holden MT, Morgan FJ, Larsen AR, Petersen A, et al A *Staphylococcus xylosus* isolate with a new *mec*C allotype. Antimicrob Agents Chemother. 2013;57(3):1524–8. 10.1128/AAC.01882-12 23274660 PMC3591899

[21] Malyszko I, Schwarz S, Hauschild T. Detection of a new *mec*C allotype, *mec*C2, in methicillin-resistant *Staphylococcus saprophyticus*. J Antimicrob Chemother. 2014;69(7):2003–5. 10.1093/jac/dku043 . 24569631

[22] Loncaric I, Kubber-Heiss A, Posautz A, Stalder GL, Hoffmann D, Rosengarten R, et al Characterization of methicillin-resistant *Staphylococcus* spp. carrying the *mec*C gene, isolated from wildlife. J Antimicrob Chemother. 2013;68(10):2222–5. 10.1093/jac/dkt186 23674764

[23] Hauschild T, Slizewski P, Masiewicz P. Species distribution of staphylococci from small wild mammals. Syst Appl Microbiol. 2010;33(8):457–60. 10.1016/j.syapm.2010.08.007 20970941

[24] Hauschild T, Stepanovic S, Zakrzewska-Czerwinska J. *Staphylococcus stepanovicii* sp. nov., a novel novobiocin-resistant oxidase-positive staphylococcal species isolated from wild small mammals. Syst Appl Microbiol. 2010;33(4):183–7. 10.1016/j.syapm.2010.03.004 20418037

[25] Jacob J, Ulrich RG, Freise J, Schmolz E (2014) Monitoring populations of rodent reservoirs of zoonotic diseases. Projects, aims and results. Bundesgesundheitsblatt Gesundheitsforschung Gesundheitsschutz 57: 511–518. 24781907 10.1007/s00103-013-1924-x

[26] Ulrich RG, Schmidt-Chanasit J, Schlegel M, Jacob J, Pelz HJ, Mertens M, et al Network "Rodent-borne pathogens" in Germany: longitudinal studies on the geographical distribution and prevalence of hantavirus infections. Parasitol Res. 2008;103 Suppl 1:S121–9. 10.1007/s00436-008-1054-9 . 19030894

[27] Hanselman BA, Kruth SA, Rousseau J, Weese JS. Coagulase positive staphylococcal colonization of humans and their household pets. Can Vet J. 2009;50(9):954–8. 19949556 PMC2726022

[28] Hyatt D, Chen GL, Locascio PF, Land ML, Larimer FW, Hauser LJ. Prodigal: prokaryotic gene recognition and translation initiation site identification. BMC bioinformatics. 2010;11:119 10.1186/1471-2105-11-119 20211023 PMC2848648

[29] Overbeek R, Olson R, Pusch GD, Olsen GJ, Davis JJ, Disz T, et al The SEED and the Rapid Annotation of microbial genomes using Subsystems Technology (RAST). Nucleic Acids Res. 2014;42(Database issue):D206–14. 10.1093/nar/gkt1226 24293654 PMC3965101

[30] Clinical and Laboratory Standards Institute (CLSI). Vet01-S2 Performance Standards for Antimicrobial Disk and Dilution Susceptibility for Bacteria isolated from Animals Second International Supplement, Wayne, PA, USA 2013;32/ 2.

[31] Clinical and Laboratory Standards Institute (CLSI). Performance standards for antimicrobial disk and dilution susceptibility tests for bacteria isolated from animals; approved standard VET01-A4. 4th ed, Wayne, PA, USA. 2013.

[32] Ito T, Ma XX, Takeuchi F, Okuma K, Yuzawa H, Hiramatsu K. Novel type V staphylococcal cassette chromosome *mec* driven by a novel cassette chromosome recombinase, *ccr*C. Antimicrob Agents Chemother. 2004;48(7):2637–51. 10.1128/AAC.48.7.2637-2651.2004 15215121 PMC434217

[33] Harrison EM, Paterson GK, Holden MT, Ba X, Rolo J, Morgan FJ, et al A novel hybrid SCC*mec*–*mec*C region in *Staphylococcus sciuri*. J Antimicrob Chemother. 2014;69(4):911–8. 10.1093/jac/dkt452 24302651 PMC3956370

[34] Tsubakishita S, Kuwahara-Arai K, Baba T, Hiramatsu K. Staphylococcal cassette chromosome mec-like element in Macrococcus caseolyticus. 2010;54(4):1469–75. 10.1128/AAC.00575-09 PMC2849352 20086147

[35] Shore AC, Deasy EC, Slickers P, Brennan G, O'Connell B, Monecke S, et al Detection of staphylococcal cassette chromosome *mec* type XI carrying highly divergent *mec*A, *mec*I, m*ec*R1, *bla*Z, and *ccr* genes in human clinical isolates of clonal complex 130 methicillin-resistant *Staphylococcus aureus*. Antimicrob Agents Chemother. 2011;55(8):3765–73. 10.1128/AAC.00187-11 21636525 PMC3147645

[36] Fessler AT, Billerbeck C, Kadlec K, Schwarz S. Identification and characterization of methicillin-resistant coagulase-negative staphylococci from bovine mastitis. J Antimicrob Chemother. 2010;65(8):1576–82. 10.1093/jac/dkq172 20525989

[37] Frey Y, Rodriguez JP, Thomann A, Schwendener S, Perreten V. Genetic characterization of antimicrobial resistance in coagulase-negative staphylococci from bovine mastitis milk. J Dairy Sci. 2013;96(4):2247–57. 10.3168/jds.2012-6091 23415536

[38] Skov R, Larsen AR, Kearns A, Holmes M, Teale C, Edwards G, et al Phenotypic detection of *mec*C-MRSA: cefoxitin is more reliable than oxacillin. J Antimicrob Chemother. 2014;69(1):133–5. 10.1093/jac/dkt341 24038776

[39] McRobbie AM, Meyer B, Rouillon C, Petrovic-Stojanovska B, Liu H, White MF. *Staphylococcus aureus* DinG, a helicase that has evolved into a nuclease. Biochem J. 2012;442(1):77–84. 10.1042/BJ20111903 22166102 PMC3270479

[40] Balakuntla J, Prabhakara S, Arakere G. Novel rearrangements in the staphylococcal cassette chromosome mec type V elements of Indian ST772 and ST672 methicillin resistant Staphylococcus aureus strains. PloS one. 2014;9(4):e94293 10.1371/journal.pone.0094293 24722327 PMC3983117

[41] Couto I, Wu SW, Tomasz A, de Lencastre H. Development of methicillin resistance in clinical isolates of *Staphylococcus sciuri* by transcriptional activation of the *mec*A homologue. J Bacteriol. 2003;185(2):645–53. 12511511 10.1128/JB.185.2.645-653.2003 PMC145312

[42] Stepanovic S, Hauschild T, Dakic I, Al-Doori Z, Svabic-Vlahovic M, Ranin L, et al Evaluation of phenotypic and molecular methods for detection of oxacillin resistance in members of the *Staphylococcus sciuri* group. J Clin Microbiol. 2006;44(3):934–7. 10.1128/JCM.44.3.934-937.2006 16517879 PMC1393147

[43] Noto MJ, Kreiswirth BN, Monk AB, Archer GL. Gene acquisition at the insertion site for SCC*mec*, the genomic island conferring methicillin resistance in *Staphylococcus aureus*. J Bacteriol. 2008;190(4):1276–83. 10.1128/JB.01128-07 18083809 PMC2238224

[44] Hiramatsu K, Ito T, Tsubakishita S, Sasaki T, Takeuchi F, Morimoto Y, et al Genomic Basis for Methicillin Resistance in Staphylococcus aureus. Infect Chemother. 2013;45(2):117–36. 10.3947/ic.2013.45.2.117 24265961 PMC3780952

[45] Everitt RG, Didelot X, Batty EM, Miller RR, Knox K, Young BC, et al Mobile elements drive recombination hotspots in the core genome of *Staphylococcus aureus*. Nat commun. 2014;5:3956 10.1038/ncomms4956 24853639 PMC4036114

[46] Chlebowicz MA, Maslanova I, Kuntova L, Grundmann H, Pantucek R, Doskar J, et al The Staphylococcal Cassette Chromosome *mec* type V from *Staphylococcus aureus* ST398 is packaged into bacteriophage capsids. Int J Med Microbiol. 2014;304(5–6):764–74. 10.1016/j.ijmm.2014.05.010 24951306

[47] Scharn CR, Tenover FC, Goering RV. Transduction of staphylococcal cassette chromosome *mec* elements between strains of *Staphylococcus aureus*. Antimicrob Agents Chemother. 2013;57(11):5233–8. 10.1128/AAC.01058-13 23939891 PMC3811280

