# Supplementary material for: A Look into the Melting Pot: The mecC-Harboring Region Is a Recombination Hot Spot in Staphylococcus stepanovicii
Source: PLoS One. 2016 Jan 22;11(1):e0147150. doi: 10.1371/journal.pone.0147150 (PMC4723332; doi:10.1371/journal.pone.0147150)
Supplement: S1 Table — Abbreviations: ORF: open reading frame, bp: base pair, C: Coverage; NI: nucleotide sequence identity; DR: direct repeat of 15 bp. 1 predicted by use of blastx. (DOCX) [file pone.0147150.s001.docx]

| **ORF** | **Description / presumptive function** | **from*** | **to*** | **bp** | **C** | **NI** | **Accession no** | **Species (gene location), strain** |
| --- | --- | --- | --- | --- | --- | --- | --- | --- |
| 1 | LSU m3Psi1915 methyltransferase RlmH (*orf*X-like gene) | 177 | 656 | 480 | 99% | 88% | HG515014.1 | *Staphylococcus sciuri* subsp. *carnaticus* (*mec* encoding region), GVGS2 |
|  | **Region between DR1 and DR2** | **639** | **10,129** | **9,419** |  |  |  |  |
| 2 | hypothetical protein | 702 | 1,526 | 825 | 97% | 69% | GQ900469.1 | *Staphylococcus epidermidis* (plasmid), SAP024A |
| 3 | hypothetical protein | 1,695 | 2,486 | 792 |  |  | no similarities |  |
| 4 | Type I restriction-modification system, restriction subunit R | 2,483 | 5,605 | 3,123 | 99% | 89% | [FR821779.1](http://www.ncbi.nlm.nih.gov/nucleotide/344176319?report=genbank&log$=nuclalign&blast_rank=1&RID=1NMN3JH701R) | *S. aureus* (type XI SCC*mec*), LGA251 |
| 5 | Type I restriction-modification system, specificity subunit S | 5,589 | 6,863 | 1,275 | 63% | 72% | [GQ902038.2](http://www.ncbi.nlm.nih.gov/nucleotide/359367790?report=genbank&log$=nuclalign&blast_rank=4&RID=BW9W7ZB8013) | *Staphylococcus aureus* (type V SCC*mec*), UMCG-M4 |
| 6 | Type I restriction-modification system, subunit M | 6,853 | 8,367 | 1,515 | 100% | 92% | CP007208.1 | *Staphylococcus xylosus,* (putative restriction modification operon), HKUOPL8 |
| 7 | hypothetical protein | 8,552 | 9,526 | 975 | 100% | 98% | [AB373032.1](http://www.ncbi.nlm.nih.gov/nucleotide/205825346?report=genbank&log$=nuclalign&blast_rank=1&RID=BWAW57CT013) | *Staphylococcus aureus*, (type 5C1 SCC*mec*), JCSC6081 |
| 8 | hypothetical protein | 9,519 | 10,067 | 549 | 100% | 91% | AB373032.1 | *Staphylococcus aureus*, (type 5C1 SCC*mec*), JCSC6081 |
|  | **Region between DR2 and DR3** | **10,130** | **14,241** | **4,112** |  |  |  |  |
| 9 | conserved domain protein | 10,395 | 11,741 | 1,347 |  |  | no similarities |  |
| 10 | hypothetical protein | 12,247 | 13,470 | 1,224 | 100% | 94% | AB705453.1 | *Staphylococcus aureus* (SCC*mec*), JCSC6690 |
| 11 | acetyltransferase | 13,635 | 14,069 | 435 | 100% | 95% | AB705453.1 | *Staphylococcus aureus* (SCC*mec*), JCSC6690 |
|  | **Region between DR3 and *orf*Y-like gene** | **14,242** | **20,707** | **6,466** |  |  |  |  |
| 12 | hypothetical protein | 14,606 | 14,761 | 156 |  |  | no similarities |  |
| 13 | hypothetical protein | 15,140 | 15,769 | 630 |  |  | no similarities |  |
| 14 | hypothetical protein | 15,835 | 16,095 | 261 |  |  | no similarities |  |
| 15 | hypothetical protein | 16,173 | 16,304 | 132 | 71% | 95% | AB705453.1 | *Staphylococcus aureus* (SCC*mec*), JCSC6690 |
| 16 | putative hypothetical protein U973_01644^1^ | 16,282 | 17,259 | 978 | 99% | 59% | EZW68885.1 | *Staphylococcus aureus,* 56864-11 |
| 17 | hypothetical protein | 17,455 | 18,930 | 1,476 | 46% | 66% | HE980450.1 | *Staphylococcus aureus* (pseudo SCC*mec*-SCC-SCCCRISPR), M06/0171 |
| 18 | putative membrane protein | 19,387 | 19,992 | 606 | 92% | 69% | CP006044.1 | *Staphylococcus aureus* CA-347, complete genome |
| 19 | acetyltransferase (GNAT) family protein | 20,147 | 20,503 | 357 | 80% | 77% | CP009554.1 | *Staphylococcus aureus*, FORC_001 |
| 20 | tRNA dihydrouridine synthase B (*orf*Y-like) | 20,708 | 21,688 | 981 | 98% | 85% | FR821777.2 | *Staphylococcus aureus*, MSHR1132 |
